# Social and Poor Law Problems

**Published:** 1906-06-30

**Authors:** 


### SOCIAL AND POOR LAW PROBLEMS.

### THE PAUPER AND HIS FOOD.

The inquiry at present going on at Poplar must have disabused the public with regard to workhouse fare being of the "cheap and nasty" order. As a matter of fact, it is probable that the food in our workhouses is better in quality and more ample in quantity than can be obtained by the poorer class of workers outside. In Stepney, which is not a specially extravagant union, the average cost of a pauper's food is about 4s. a week, and as guardians, buying in bulk, can make a better bargain—if they are honest towards the ratepayers and make their purchases in the best market—this means more than the same sum spent in a private household. The cost of the pauper's clothing is set down at Stepney at 5d. a week on an average. As the reports of many other unions do not distinguish between the cost of food and of clothing, both these items being set down under "maintenance," it may be convenient to deduct 5d. a week from the total cost for each pauper. Thus in Wandsworth we find that maintenance and clothing are reckoned at less than a halfpenny more than Stepney. In Whitechapel, however, the amount spent on food and necessaries for the inmates of the workhouse is only 3s. 7¼d. per head per week, and clothing, here differentiated, amounts only to 2⅝d. per week. The difference in expenditure between two parishes so near each other and so similar in many respects as Stepney and Whitechapel is rather remarkable, and shows how wide a latitude is allowed to local authorities. We have not the detailed dietaries of Stepney, but as the cost of food in Wandsworth is almost the same as the East End union, we may compare the food given there with that in Whitechapel, with a view to discovering how the latter manages to feed the inmates of its workhouse for less.

Sunday breakfast is much the same in all workhouses—1 pint of tea, 8 oz. of bread, and ½ oz. margarine for men, and 6 oz. of bread for women. Supper is the same as breakfast. Dinner consists of 4½ oz. of roast beef (or alternatively in Whitechapel of boiled mutton), with 12 oz. of potatoes and other vegetables. Children have a smaller quantity of meat, and in Wandsworth have instead 8 oz. of milk pudding. In Bethnal Green workhouse the Sunday dinner is of cold meat, and in Islington certain classes, both of men and women, receive only coffee with bread and cheese for dinner on Sunday, while the rest of the inmates are regaled with roast mutton; but one may take it as a general rule that on Sunday at least the pauper's fare is such as no one either inside or outside a workhouse need reasonably complain of.

On weekdays differences begin. Lambeth gives its workhouse inmates 4½ oz. of boiled beef and 12 oz. of potatoes for dinner, and Wandsworth 3½ oz. of boiled mutton with 6 oz. of potatoes and other vegetables, and 4 oz. of suet dumpling, while in Hackney the pauper of the same class (able-bodied male) gets only 1½ pint of soup. He has, however, 6 oz. of bread at that meal, while in the unions previously mentioned the pauper receives only 4 oz. at dinner. Still, with his meat and suet pudding he is decidedly better off. In Southwark the pauper has the option of having bread and cheese with coffee instead of his 1½ pint of pea or barley soup. In Hampstead Monday's dinner consists of 3 oz. of boiled bacon, but both Lewisham and Whitechapel are faithful to the soup diet, while in Bethnal Green the pauper's dinner consists of 16 oz. of suet pudding and ½ pint of stewed fruit, but he has a pint of broth as well as bread and cheese for his supper, while in most of the other unions gruel for the men and tea for the women is the rule. In Lambeth, however, broth is given for supper, although the inmate has had a pretty good dinner. Lambeth also gives its able-bodied men and women—who, of course, are given work to do—a lunch of bread and cheese at 10 A.M., but children there get only bread and dripping for lunch, while in Wandsworth they are fortunate in having a piece of plain or seed cake. One cannot help thinking that paupers must have good appetites, when one finds that in Wandsworth Wednesday's dinner consists of 16 oz. of meat pudding, with 6 oz. of vegetables and 4 oz. of bread, while in Kensington on Thursday the pauper has 16 oz. of meat pie, but apparently neither bread nor vegetables. In many unions fish is given for dinner on Fridays. This is a wholesome change, and doubtless the selection of Friday for the fish dinner is due to consideration for the scruples of those whose religion makes regulations for their diet. This consideration for the feelings of the inmates deserves to be noted. It is specifically mentioned in the regulations for each union that the quantities of meat mentioned mean cooked meat, free from bone (excepting in the case of fish). If an inmate should be doubtful whether or not he has received his full allowance, he is within his rights if he demands that the food should be weighed in his presence and that of the master of the workhouse, and if there is a deficiency have it made up. The ingredients and the amounts of each to be used in the making of soup, hash, Irish stew, suet or meat pudding, in the cake and the milk puddings provided for children, and in the tea, coffee, and cocoa which the majority of the inmates have for supper, is fixed by tables, which must be approved by the Local Government Board. The diet-sheets also must be approved by that body; but, as will be inferred from the slight sketch which we have given of workhouse fare in London, uniformity is not insisted on, and Boards of Guardians can, within limits, be as generous as they like to their wards. On the whole they are by no means harsh. To give tables of all the Metropolitan workhouse dietaries would be uninteresting, but their nature may be inferred from the examples given. Everywhere women get rather less meat than men, and children still smaller quantities, varying according to their age, but for compensation they have milk pudding daily. Their breakfast consists of bread and milk or porridge, and their supper of milk or tea, with bread and margarine or jam.

Besides the regulation diets some inmates—the old, the infirm, and those who do extra work—get special consideration—tobacco for the men and extra tea for the old women. They are very jealous of their rights in these matters, and if, because of some infraction of discipline or other reason, they are for a time deprived of their privileges, they are pretty sure to demand to see the guardians and either give or demand an explanation. The present writer remembers an old dame who complained to the guardians who were visiting the workhouse where she was boarded—her own union being crowded—that she had not "the necessaries of life." This naturally led to inquiry, in the course of which it transpired that the "necessaries of life" alluded to were tea and sugar. The complainant admitted that she had tea for breakfast and supper, and finally explained that she had recently been deprived of her "cup of tea after dinner, which she always enjoyed very much." Happily the deprivation was only a temporary one, due to her having gone out on a day other than the regulation visiting day, which compelled the authorities to register her as a new-comer when she returned, and the afternoon tea which she missed was given only to those who had been a month in the institution.

While the amount of food given to paupers is checked by the Local Government Board, the quality of it depends very largely upon the guardians. But the specifications for goods always demand a good quality of article, and if the goods supplied are not up to contract and sample, the master or steward of the institution has, as a rule, little hesitation in refusing to accept it. It is not needful that the most expensive quality of everything should be supplied, though some boards seem to think that what is good enough for the free worker is not good enough for the pauper. But the tendency is certainly to make sure that the food supplied is good as well as sufficient; and it is doubtful if the poorer class of workers fare as well outside as the inmate of the workhouse does.

